# T cells in the brain may contribute to attenuation of sepsis-associated depression

**DOI:** 10.1038/s42003-021-01923-7

**Published:** 2021-03-17

**Authors:** Karli Montague-Cardoso

**Affiliations:** Communications Biology, https://nature.com/commsbio

## Abstract

Sepsis-associated encephalopathy, as well as increasing mortality, has been associated with long-lasting depressive behaviour, which is thought to be caused by infection-induced neuroinflammation in the brain. Saito et al. have recently demonstrated in a mouse model of sepsis that infiltrated regulatory T cells in the cerebral cortex mediate the resolution of neuroinflammation and alleviate anxious/depressive behaviour. Their study paves the way for further research that investigates the role of T cells in the underlying mechanisms mediating recovery of sepsis-associated depression.

**Figure:**
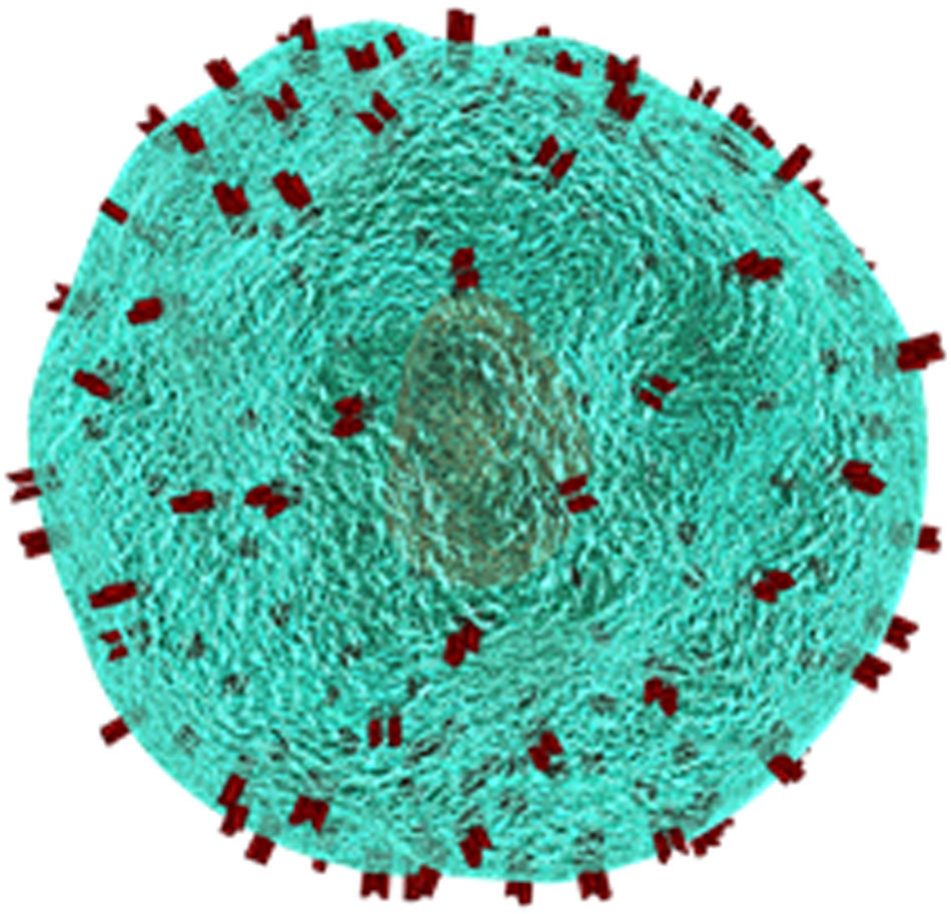
Pixabay

Sepsis can lead to encephalopathy, which as well as being associated with an increase in both morbidity and mortality, is also often associated with depression and anxiety. We know from recent studies that such cognitive effects are likely to be the result of neuroinflammation caused by the infection. However a more precise underlying mechanism had yet to be firmly established and the specific involvement of T cells remained unclear.

In a recent study, Saito et al.^1^ used a mouse model of sepsis to assess the role of T cells in the mediation of both the development and recovery of sepsis-associated depression. They used a batch of behavioural tests to assess cognitive state in septic mice in conjunction with flow cytometry, quantitative real-time PCR and immunohistochemistry to examine neuroinflammation as well as quantify and characterise infiltrated T cells in the cerebral cortex at different time points. They report that within about a week of sepsis onset, mice displayed anxious behaviour and T cells had infiltrated into the brain. By about a month post-sepsis onset however, neuroinflammation had resolved, as indicated by levels of inflammatory cytokines such as TNFa, returning to normal, physiological levels. Interestingly, Saito et al. found that the resolution of inflammation as well as improvement in depressive behaviour, was significantly delayed when mice were also treated with FTY720, which inhibits immune cell egression from lymph nodes. This therefore indicates that infiltrated immune cells play a key role in inflammation and anxiety resolution. When Saito et al. went on to characterise the infiltrated T cells using flow cytometry, they observed that the infiltrating T cells were regulatory T cells (Tregs) as well as Th2 cells in untreated, septic mice, however in FTY720-treated mice, the T cell population in the cortex were predominantly Th17 cells. This strongly suggests that the resolution of neuroinflammation and recovery from depression is mediated specifically by infiltrated Tregs and Th2 cells.

Taken together the findings reported in this study effectively demonstrate a potential role for infiltrated Tregs and Th2 cells in the resolution of sepsis-induced inflammation and its associated anxiety/depression. This provides some important groundwork that warrants further investigation into the mechanistic role of T cells in this context, which could open up a new avenue for investigating potential therapeutic targets.

## References

[1] Saito M (2021). Infiltrated regulatory T cells and Th2 cells in the brain contribute to attenuation of sepsis-associated encephalopathy and alleviation of mental impairments in mice with polymicrobial sepsis. Brain Behav. Immun..

